# Protocol for analyzing tumor-infiltrating bystander CD8^+^ T cells in mice using adoptive transfer of P14 TCR-transgenic CD8^+^ T cells

**DOI:** 10.1016/j.xpro.2026.104886

**Published:** 2026-10-06

**Authors:** Chunyang Xie, Wenwen Xi, Hairu Wang, Lilin Ye, Xiangyu Chen, Yao Lin

**Affiliations:** 1 Guangdong Province Key Laboratory of Immune Regulation and Immunotherapy, School of Laboratory Medicine and Biotechnology, Southern Medical University, Guangzhou, Guangdong 510515, China; 2 Institute of Immunology, Army Medical University (Third Military Medical University), Chongqing 400038, China; 3 Department of Rheumatology and Immunology, Center for Immune Ageing and Rejuvenation, The First Affiliated Hospital of Chongqing Medical University, Chongqing 400016, China; 4 Department of Rheumatology and Immunology, Center for Diagnosis and Treatment of Rare and Complex Diseases, The Third People’s Hospital of Chengdu, Chengdu 610031, China

**Keywords:** Cell isolation, Flow Cytometry, Cancer, Immunology, Model Organisms

## Abstract

While tumor-specific CD8^+^ T cells become functionally exhausted within the tumor microenvironment (TME), virus-specific bystander memory CD8^+^ T (T_BYS_) cells are abundant and represent valuable immunotherapy targets. Here, we present a protocol to analyze tumor-infiltrating virus-specific T_BYS_ cells in murine tumor models using P14 CD8^+^ T cells. We describe steps for monitoring long-term *in vivo* behavior of P14 T_BYS_ cells and characterizing their differentiation trajectories and functional heterogeneity.

For complete details on the use and execution of this protocol, please refer to Lin et al.^1^

## Highlights

• Steps for setting up mouse model and tracking tumor-infiltrating bystander CD8^+^ T cells
• Instructions for generating LCMV-specific memory P14 CD8^+^ T cells
• Steps for establishing subcutaneous tumor models
• Guidance on flow cytometric phenotyping of tumor-infiltrating bystander CD8^+^ T cells

## Abstract (editor highlights)

Publisher’s note: Undertaking any experimental protocol requires adherence to local institutional guidelines for laboratory safety and ethics.

## Graphical abstract

**Figure:**
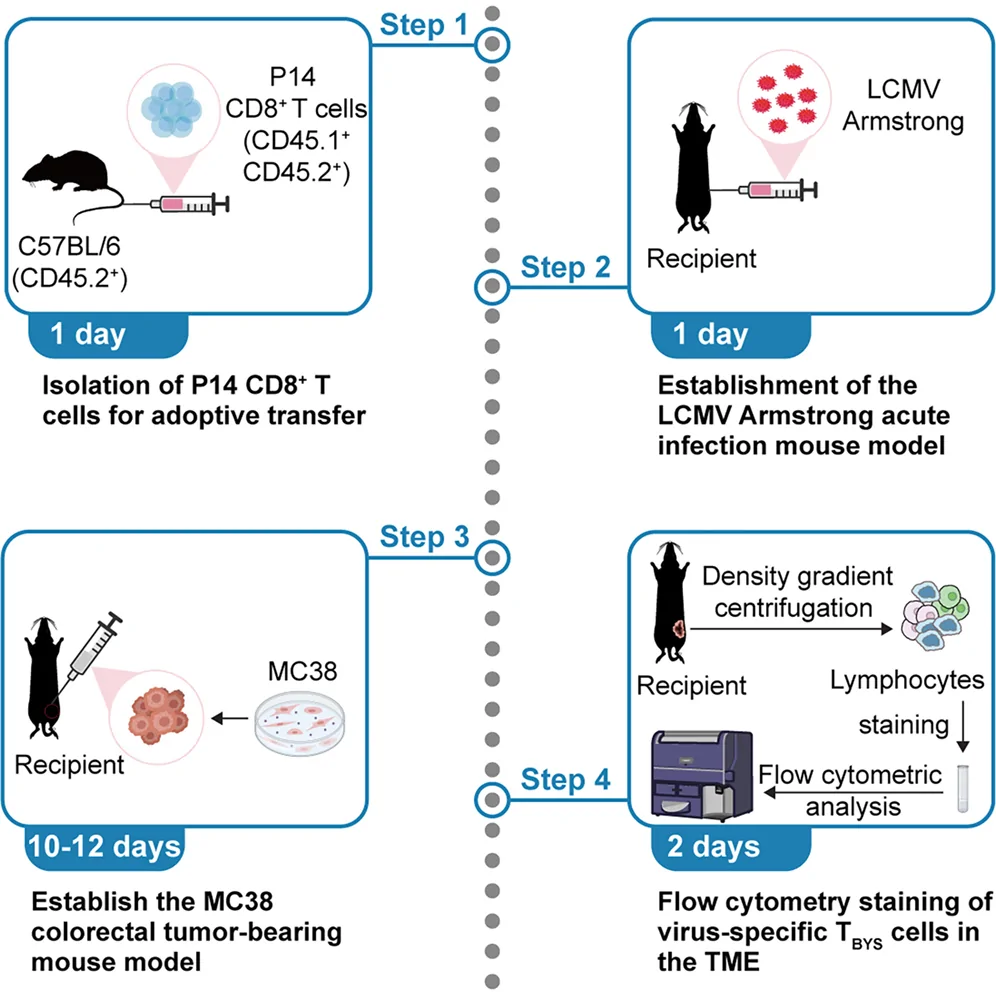

## Before you begin

Following acute viral infection or vaccination, virus-specific CD8^+^ T cells differentiate into long-lived memory T (T_MEM_) cells to establish durable protective immunity against viral rechallenge.^2^^,^^3^ By contrast, persistent tumor antigen stimulation drives tumor antigen-specific CD8^+^ T cells toward functional exhaustion,^4^ and the resulting failure of these exhausted cells to acquire T_MEM_ properties markedly blunts therapeutic responses to immunotherapy, as exemplified by PD-1 immune checkpoint blockade.^5^ However, emerging evidence demonstrates that virus-specific T_MEM_ cells primed by either natural acute infection or vaccination are abundantly recruited into the tumor microenvironment (TME).^6^^,^^7^^,^^8^ These TME-infiltrating virus-specific T_MEM_ cells do not recognize tumor antigens and are therefore defined as bystander memory CD8^+^ T (T_BYS_) cells.

### Innovation

Our group and others have demonstrated that virus-specific T_BYS_ cells can be exploited to elicit anti-tumor immunity, highlighting their potential as a therapeutic avenue for cancer treatment.^9^^,^^10^ However, the mechanisms governing the differentiation and maintenance of virus-specific T_BYS_ cells within the TME remain poorly understood. The protocol described herein provides a practical approach for dissecting the differentiation trajectories, functional properties, and underlying regulatory networks of virus-specific T_BYS_ cells within the TME.

### Institutional permissions

All mouse experiments were carried out in accordance with procedures approved by the Institutional Animal Care and Use Committees of the Third Military Medical University (Approval No. AMUWEC2023674).

All animal procedures require prior approval from the institutional animal care and use committee to ensure regulatory compliance.

### Preparation: Breeding and genotyping of P14 mice (CD45.1^+^CD45.2^+^)

**Timing: Over 12 weeks**

1. Cross C57BL/6 mice on a CD45.1 background with P14 TCR transgenic mice on a CD45.2 background.

***Note:*** For adoptive transfer assays, P14 mice are backcrossed to a CD45.1 congenic background to enable unambiguous discrimination of donor and recipient cells via CD45.1/CD45.2 congenic markers.

2. Phenotype P14 (CD45.1^+^CD45.2^+^) mice by flow cytometry.
  a. Collect ∼100 μL (3 drops) of peripheral blood via tail tip excision into 1 mL red blood cell (RBC) lysis buffer.
  b. Centrifuge samples at 800 × *g* for 5 min and discard supernatant. Resuspend cells in 200 μL of FACS buffer (see materials and equipment for buffer recipe) and transfer to a round-bottom 96-well plate. Keep the plate on ice.
  c. Prepare antibody cocktail in 50 μL FACS buffer per sample.
    ***Note:*** P14 cells are defined as Vα2^+^Vβ8^+^ cells within the CD8^+^ T cell compartment; the surface staining cocktail includes antibodies against Vα2, Vβ8, CD8, CD45.1, CD45.2, CD44, and Live/Dead dye. The antibody staining cocktail is detailed in the materials and equipment section.
  d. Incubate samples with the prepared antibody cocktail.
    i. Centrifuge the 96-well plate at 800 × *g* for 1 min and discard supernatant by decantation.
    ii. Resuspend cell pellets in 50 μL FACS buffer containing Fc-Block (1:200 final dilution) and incubate on ice for 20 min.
    iii. Add 50 μL pre-prepared antibody cocktail directly to each well.
    iv. Resuspend thoroughly by gentle pipetting and incubate on ice for 30 min protected from light.
  e. Wash cells twice with FACS buffer and transfer to flow cytometry tubes for acquisition.
    i. Add 150 μL FACS buffer to each well, centrifuge at 800 × *g* for 1 min, discard the supernatant by decantation, and briefly vortex the plate.
    ii. Add 200 μL FACS buffer to each well, centrifuge at 800 × *g* for 1 min, discard the supernatant by decantation, and briefly vortex the plate.
    iii. Resuspend cells in 200 μL of FACS buffer per well and transfer to flow cytometry tubes.
  f. Acquire samples on a BD Fortessa flow cytometer (Figure S1).
    i. Gate Live/Dead^−^CD8^+^CD44^−^ cells as naïve live CD8^+^ T cells.
    ii. Gate Vα2^+^Vβ8^+^ cells within the naïve CD8^+^ T cell gate as P14 cells.
    iii. Gate CD45.1^+^CD45.2^+^ cells within the P14 gate to confirm congenic marker expression.
      ***Note:*** Mice with ≥80% Vα2^+^Vβ8^+^ cells among naïve live CD8^+^ T cells are confirmed as P14 transgenic positive.

## Key resources table

**Table:** 

REAGENT or RESOURCE	SOURCE	IDENTIFIER
**Antibodies**		

PerCP/Cyanine5.5 anti-mouse CD8a (clone 53-6.7)	Biolegend	Cat# 100734; RRID: AB_2075238 Working dilution: 1:400
Brilliant Violet 510™ anti-mouse CD8a (clone 53-6.7)	Biolegend	Cat# 100752; RRID: AB_2563057 Working dilution: 1:400
PerCP/Cyanine5.5 anti-mouse CD44 (clone IM7)	Biolegend	Cat# 103032; RRID: AB_2076204 Working dilution: 1:400
APC anti-mouse CD45.1 (clone A20)	Biolegend	Cat# 110713; RRID: AB_313502 Working dilution: 1:400
PE/Cyanine7 anti-mouse CD62L (clone MEL-14)	Biolegend	Cat# 104418; RRID: AB_313103 Working dilution: 1:400
Brilliant Violet 421™ anti-mouse CD127 (IL-7Rα) (clone A7R34)	Biolegend	Cat# 135027; RRID: AB_2563103 Working dilution: 1:400
PE anti-mouse CD69 (clone H1.2F3)	Biolegend	Cat# 164203; RRID: AB_2936560 Working dilution: 1:400
FITC anti-mouse CD69 (clone H1.2F3)	Biolegend	Cat# 104505; RRID: AB_313108 Working dilution: 1:400
PerCP anti-mouse CD45.1 (clone A20)	Biolegend	Cat# 110725; RRID: AB_893347 Working dilution: 1:400
PE anti-mouse TCR Vβ8 (clone KJ16-133.18)	Biolegend	Cat# 118408; RRID: AB_1134109 Working dilution: 1:400
PE anti-mouse/human KLRG1 (MAFA) (clone 2F1)	Biolegend	Cat# 138407; RRID: AB_10574005 Working dilution: 1:400
Alexa Fluor® 700 anti-mouse CD45.2 (clone 104)	Biolegend	Cat# 109822; RRID: AB_493731 Working dilution: 1:400
Pacific Blue™ anti-mouse CD45.2 (clone 104)	Biolegend	Cat# 109819; RRID: AB_492873 Working dilution: 1:400
Alexa Fluor® 647 anti-mouse TCR Vα2 (clone B20.1)	Biolegend	Cat# 127812; RRID: AB_1186115 Working dilution: 1:400
FITC anti-mouse CD103 (clone 2E7)	Biolegend	Cat# 121420; RRID: AB_10714791 Working dilution: 1:200
Brilliant Violet 605™ anti-mouse CX3CR1 (clone SA011F11)	Biolegend	Cat# 149027; RRID: AB_2565937 Working dilution: 1:400
TCF1/TCF7 Rabbit Monoclonal Antibody (Alexa Fluor® 488 Conjugate) (clone C63D9)	Cell Signaling Technology	Cat# 6444S; RRID: N/A Working dilution: 1:200
Rat anti-Mouse CD16/CD32 mAb (Mouse Fc-Block)	ABclonal	Cat# A29013; RRID: N/A Working dilution: 1:200
Biotin anti-mouse/human CD45R/B220 Antibody (clone RA3-6B2)	Biolegend	Cat# 103204; RRID: AB_312989 Working dilution: 1:400
Biotin anti-mouse NK-1.1 Antibody (clone PK136)	Biolegend	Cat# 108704; RRID: AB_313391 Working dilution: 1:500
Biotin anti-mouse F4/80 Antibody (clone BM8)	Biolegend	Cat# 123106; RRID: AB_893501 Working dilution: 1:500
Biotin anti-mouse TER-119/Erythroid Cells Antibody (clone TER-119)	Biolegend	Cat# 116204; RRID: AB_313705 Working dilution: 1:500
Biotin anti-mouse CD11c Antibody (clone N418)	Biolegend	Cat# 117304; RRID: AB_313773 Working dilution: 1:500
Biotin anti-mouse/human CD44 Antibody (clone IM7)	Biolegend	Cat# 103004; RRID: AB_312955 Working dilution: 1:400
Biotin anti-mouse CD4 Antibody (clone RM4-5)	Biolegend	Cat# 100508; RRID: AB_312711 Working dilution: 1:400

**Bacterial and virus strains**		

LCMV Armstrong	Dr. Rafi Ahmed	N/A

**Chemicals, peptides, and recombinant proteins**		

Trypsin-EDTA (0.25%), phenol red	Gibco	Cat# 25200056
Fetal Bovine Serum (FBS)	Gibco	Cat# A5669701
RPMI 1640	Gibco	Cat# 11875093
Dulbecco’s Modified Eagle Medium (DMEM)	Gibco	Cat# 11965092
L-Glutamine 200mM (100×)	Gibco	Cat# 25030081
Non-Essential Amino Acids (MEM NEAA) (100×)	Gibco	Cat# 11140050
Sodium Pyruvate (100mM)	Gibco	Cat# 11360070
Penicillin-Streptomycin-Glutamine Solution	Gibco	Cat# 10378016
Percoll	GE Healthcare	Cat# 17-0891-09
Collagenase Type I	Diamond	Cat# A004194
BD IMag™ Streptavidin Particles Plus - DM	BD Bioscience	Cat# 557812
Calcium chloride	Beyotime	Cat# Y001521
Magnesium chloride	Beyotime	Cat# Y001529
DNase I	Beyotime	Cat# D7076
10 × PBS	Life-iLab	Cat# AC08L022
1 × PBS	Biosharp	Cat# BL302A
Red blood cell lysis buffer	Beyotime	Cat# C3702

**Critical commercial assays**		

Foxp3/Transcription Factor Staining Buffer Kit	eBioscience	Cat# 00–5523
LIVE/DEAD Fixable Near-IR Dead Cell Stain Kit	Thermofisher	Cat# L34975 Working dilution: 1:400

**Experimental models: Cell lines**		

MC38 cells (*Mus musculus*, colon adenocarcinoma)	Shanghai Model Organisms Center	Cat# NM-S13

**Experimental models: Organisms/strains**		

Mouse: C57BL/6J (CD45.2), 8-week-old, sex-matched (male or female)	Jackson Laboratory	Stock No: 000664
Mouse: B6.Cg-Tcra^tm1Mom^ Tg (TcrLCMV) 327Sdz/TacMmjax (P14), 8-week-old donors, sex-matched with recipients	From Dr. Rafi. Ahmed (Emory University)	N/A

**Software and algorithms**		

FlowJo	BD Bioscience	version 10.9.0 https://www.flowjo.com RRID: SCR_008520

**Other**		

70 μm cell strainer	Biosharp	Cat# BS-70-CS
Round-bottom 96-well plate	Beyotime	Cat# FPT016
BD IMag™ Cell Separation Magnet	BD Bioscience	Cat# 552311

## Materials and equipment

**Table:** FACS buffer

Reagent	Final concentration	Amount
FBS	2 %	1 mL
Sodium azide (NaN_3_)	0.1 mg/mL	5 mg
PBS	N/A	49 mL
Total	N/A	supplement to 50 mL

Store at 4°C for up to 1 month.

**Table:** P14 genotyping surface-staining cocktail

Reagent	Dilution	Amount (per sample)
anti-mouse TCR Vα2 Antibody	1:400	0.125 μL
anti-mouse TCR Vβ8 Antibody	1:400	0.125 μL
anti-mouse CD8a Antibody	1:400	0.125 μL
anti-mouse CD45.1 Antibody	1:400	0.125 μL
anti-mouse CD45.2 Antibody	1:400	0.125 μL
anti-mouse CD44 Antibody	1:400	0.125 μL
Live/Dead dye	1:400	0.125 μL
FACS buffer	N/A	supplement to 50 μL

Prepare fresh on the day of staining; keep protected from light on ice.

**Table:** Biotinylated antibody cocktail for naïve CD8^+^ T cell enrichment

Reagent	Dilution	Amount (per 1 × 10^8^ cells)
Biotin anti-mouse CD4 Antibody	1:400	2.5 μL
Biotin anti-mouse CD44 Antibody	1:400	2.5 μL
Biotin anti-mouse CD19 Antibody	1:400	2.5 μL
Biotin anti-mouse NK1.1 Antibody	1:500	2 μL
Biotin anti-mouse F4/80 Antibody	1:500	2 μL
Biotin anti-mouse CD11c Antibody	1:500	2 μL
Biotin anti-mouse Ter119 Antibody	1:500	2 μL
PBS	N/A	supplement to 1 mL

Prepare fresh on the day of use; keep on ice.

**Table:** D10 medium

Reagent	Final concentration	Amount
FBS	10 %	5 mL
Non-essential amino acids	1 %	500 μL
Sodium pyruvate	1 %	500 μL
Penicillin/streptomycin	1 %	500 μL
DMEM	N/A	43.5 mL
Total	N/A	50 mL

Store at 4°C for up to 1 month.

**Table:** Tumor digestion solution

Reagent	Final concentration	Amount
FBS	2 %	1 mL
Collagenase Type I	150 U/mL	60 mg
Calcium chloride (CaCl_2_)	1 mM	50 μL (stock 1 M)
Magnesium chloride (MgCl_2_)	1 mM	50 μL (stock 1 M)
DNase I	50 U/mL	125 μL (stock 10 mg/mL)
RPMI-1640 medium	N/A	supplement to 50 mL
Total	N/A	50 mL

Prepare fresh on the day of use, maintain at 20°C–25°C prior to use.

**Table:** Tumor-infiltrating virus-specific T_BYS_ cells surface staining cocktail

Reagent	Dilution	Amount (per sample)
anti-mouse CD8a Antibody	1:400	0.125 μL
anti-mouse CD45.1 Antibody	1:400	0.125 μL
anti-mouse CD44 Antibody	1:400	0.125 μL
anti-mouse KLRG1 Antibody	1:400	0.125 μL
anti-mouse CD127 Antibody	1:400	0.125 μL
anti-mouse CD69 Antibody	1:400	0.125 μL
anti-mouse CD62L Antibody	1:400	0.125 μL
anti-mouse CX3CR1 Antibody	1:400	0.125 μL
anti-mouse CD103 Antibody	1:200	0.25 μL
Live/Dead dye	1:400	0.125 μL
FACS buffer	N/A	supplement to 50 μL

Prepare fresh on the day of staining; keep protected from light on ice.

**Table:** Tumor-infiltrating virus-specific T_BYS_ cells intracellular-nuclear staining cocktail

Reagent	Dilution	Amount (per sample)
TCF1/TCF7 Rabbit Monoclonal Antibody	1:200	0.25 μL
1× permeabilization buffer	N/A	supplement to 50 μL

Prepare fresh on the day of staining; keep protected from light on ice.

**Table:** 44 % Percoll solutions (single-sample scale)

Reagent	Final concentration	Amount
Percoll	44 %	3.168 mL
10 × PBS	1 × PBS	0.352 mL
RPMI 1640 medium	N/A	4.480 mL

Prepare fresh on the day of use, equilibrate to 20°C–22°C before density-gradient setup.

**Table:** 67 % Percoll solutions (single-sample scale)

Reagent	Final concentration	Amount
Percoll	67 %	2.412 mL
10 × PBS	1 × PBS	0.268 mL
1 × PBS	N/A	1.320 mL

Prepare fresh on the day of use, equilibrate to 20°C–22°C before density-gradient setup.

## Step-by-step method details

### Isolation of P14 CD8^+^ T cells for adoptive transfer

**Timing: 1 day**

This section describes isolation of naïve P14 CD8^+^ T cells from donor mouse spleens for adoptive transfer (Figure 1).

**CRITICAL:** Strict sex-matching between donor and recipient mice is required for this adoptive-transfer experiment. Use male-to-male or female-to-female transfers only, as sex-related biological differences may alter T-cell engraftment and tumor-infiltration outcomes.

1. Euthanize 8-week-old CD45.1^+^CD45.2^+^ P14 TCR transgenic donor mice in accordance with institutional animal care guidelines.
2. Harvest spleens and generate single-cell lymphocyte suspensions.
  a. Create a 1-cm incision in the left peritoneal wall using sterile surgical scissors.
  b. Excise the spleen and carefully remove adherent connective tissue.
  c. Place the spleen on a 70 μm cell strainer seated in a petri dish, and mechanically dissociate tissue through the strainer using a syringe plunger with 3 mL RBC lysis buffer. Incubate the cell suspension at 20°C–25°C for 3–5 min to lyse red blood cells.
  d. Add 6 mL of RPMI 1640 supplemented with 2% FBS (RPMI-2%) to the dish to dilute RBC lysis buffer.
  e. Transfer cell suspensions from the dish to a 15 mL conical tube.
  f. Centrifuge cell suspensions at 800 × *g* for 5 min and discard the supernatant by decantation.
  g. Resuspend cells with RPMI-2% buffer, adjust the cell density to ∼1 × 10^8^ cells per mL and maintain on ice.

***Note:*** A typical 8-week-old P14 TCR-transgenic mouse yields 0.5–1.5 × 10^8^ total splenocytes from one spleen; naïve P14 CD8^+^ T cells account for approximately 10%–20% of total splenocytes.

3. Perform negative magnetic selection to enrich naïve CD8^+^ T cells.
  a. Prepare 1 × biotinylated antibody cocktail against CD4, CD44, CD19, NK1.1, F4/80, CD11c, and Ter119 in PBS for naïve CD8^+^ T cell enrichment (see materials and equipment for full cocktail composition).
  b. Centrifuge cell suspensions at 800 × *g* for 5 min and discard supernatant by decantation.
  c. Resuspend cells in 1 mL of the biotinylated antibody cocktail (for up to 1 × 10^8^ total cells) in a 1.5 mL microcentrifuge tube, and incubate on ice with gentle agitation for 30 min.
  d. Wash cells by centrifugation at 800 × *g* for 5 min and discard supernatant.
  e. Resuspend cell pellets in 1 mL PBS in a 1.5 mL microcentrifuge tube, then add 150 μL streptavidin-conjugated magnetic beads suspension (for up to 1 × 10^8^ total cells), and incubate with gentle continuous end-over-end rotation on a rotator at 4°C for 40 min.
  f. Place the tube on a magnetic separation stand for 4 min until all beads are bound to the magnet-facing tube wall.
  g. Carefully collect the unbound supernatant (containing enriched CD8^+^ T cells) without disturbing the magnetic-bead pellet, and transfer to a new 15 mL centrifuge tube.
  h. Centrifuge cell suspensions at 800 × *g* for 5 min and discard supernatant by decantation.
  i. Resuspend cells in 2 mL of RPMI-2% buffer and maintain on ice.
4. Phenotype and quantify purified P14 cells by flow cytometry.
  a. Aliquot 50 μL cell suspension to a 96-well plate and add 150 μL FACS buffer.
  b. Centrifuge samples at 800 × *g* for 2 min and discard supernatant.
  c. Resuspend cell pellets in 50 μL FACS buffer containing Fc-Block (1:200 final dilution) and incubate on ice for 20 min.
  d. Add 50 μL antibody cocktail (identical to Preparation step 2c) directly to each well and incubate on ice for 30 min protected from light.
  e. Wash cells twice with FACS buffer and transfer to flow cytometry tubes for acquisition.
  f. Calculate P14 CD8^+^ T cell yield for adoptive transfer.
    i. Gate Vα2^+^Vβ8^+^ cells within naïve live CD8^+^ T cells as P14 CD8^+^ T cells.
    ii. Record the frequency of P14 CD8^+^ T cells among total lymphocytes. Calculate the absolute number of P14 CD8^+^ T cells by multiplying this frequency by the total viable cell count obtained using a hemocytometer.
    iii. Wash the purified naïve P14 CD8^+^ T cells twice with serum-free RPMI medium prior to adoptive transfer.

***Note:*** Following this enrichment protocol, the typical purity of naïve P14 CD8^+^ T cells ranges from 80 %–95 %, with a typical yield of 4–12 × 10^6^ P14 CD8^+^ T cells per donor mouse spleen.

5. Adoptively transfer P14 CD8^+^ T cells to recipient mice.
  a. Warm 8-week-old C57BL/6 recipient mice (CD45.2^+^) under an infrared lamp to dilate tail veins for intravenous injection.
  b. Draw up 100 μL cell suspension (in serum-free RPMI, containing 5 × 10^4^ purified P14 CD8^+^ T cells) into an insulin syringe.
    ***Note:*** Centrifuge cells and wash twice in serum-free RPMI prior to transfer to remove residual FBS.
  c. Insert the syringe needle into the tail vein and inject the cell suspension at a slow, steady rate.

**Figure 1:**
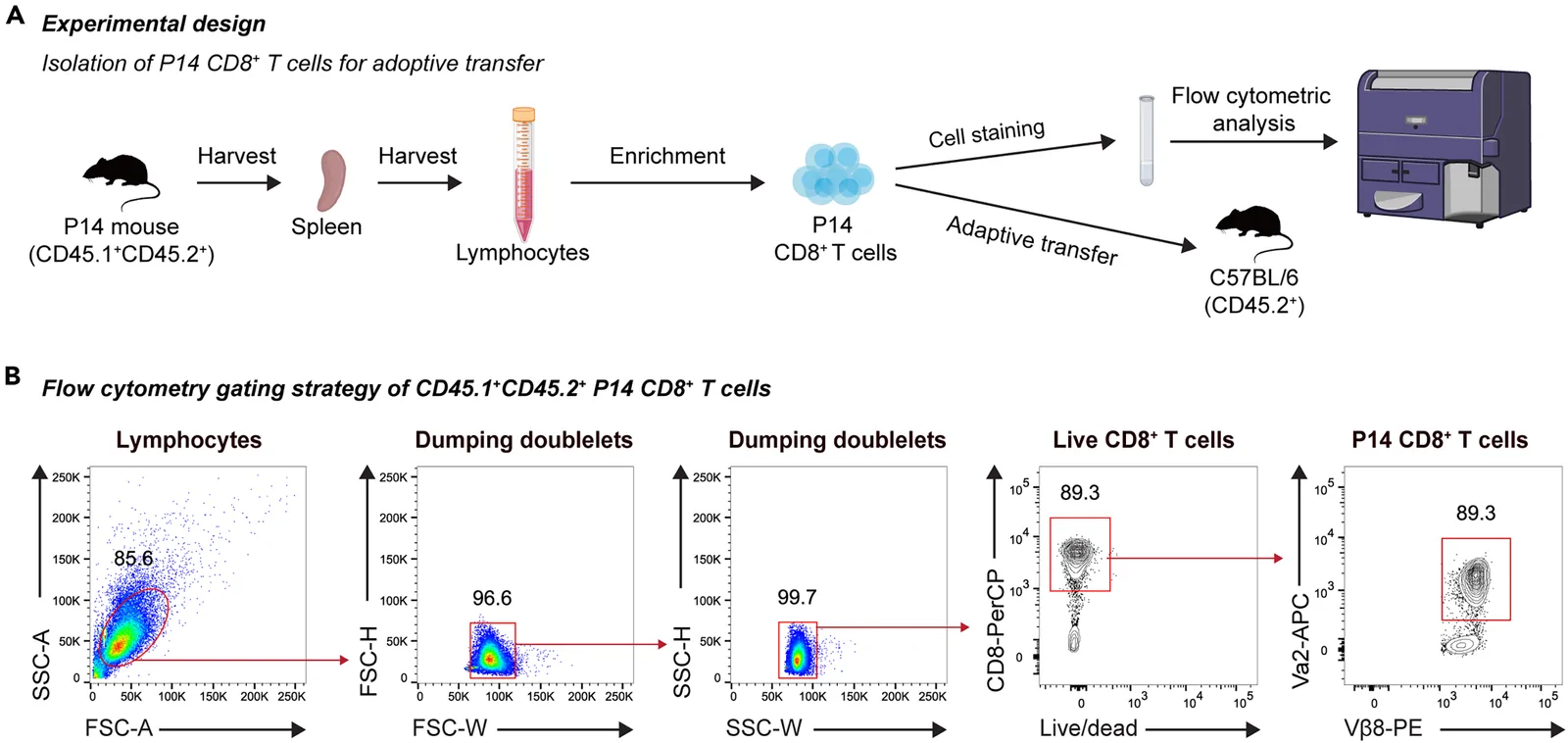
Isolation of naïve P14 CD8^+^ T cells from murine spleens for adoptive transfer (A) Schematic overview of the experimental workflow. Harvest spleens from P14 mice to generate lymphocyte suspensions. Purify naïve P14 CD8^+^ T cells and stain an aliquot for flow cytometry, then gate Vα2^+^Vβ8^+^ cells to calculate absolute P14 cell counts. Finally, inject quantified P14 CD8^+^ T cells into CD45.2^+^ C57BL/6 mice. (B) Representative flow cytometry plots showing the gating strategy for splenic P14 CD8^+^ T cells from donor mice.

### Establishment of acute LCMV Armstrong infection

**Timing: 1 day**

This section describes induction of acute LCMV Armstrong infection to generate memory P14 CD8^+^ T cells.

6. One day after adoptive transfer, dilute LCMV Armstrong virus stock in RPMI to a final concentration of 1 × 10^6^ plaque-forming units (PFU) per mL.
7. Inject 100 μL diluted virus (containing 1 × 10^5^ PFU) intraperitoneally into each recipient mouse.

### Phenotypic analysis of infection-induced memory P14 CD8^+^ T cells

**Timing: 1 day**

This section describes isolation, staining, and flow cytometric analysis of virus-specific memory CD8^+^ T cells (Figure 2).

8. Two months post-infection, isolate peripheral blood lymphocytes and perform flow cytometric staining.
  a. Collect ∼100 μL peripheral blood via tail tip excision into 1 mL RBC lysis buffer, and incubate the mixture for 5 min at 22°C–25°C for RBC lysis.
    ***Note:*** This approach reliably yields sufficient blood volume for downstream flow cytometric analysis, especially for repeated sampling from mice with limited peripheral cell numbers. While tail-vein sampling is a less-invasive alternative, it often yields insufficient sample volume for this specific assay in our hands. For multiple parallel samples, collect blood into EDTA-coated tubes first to prevent clotting before adding RBC lysis buffer.
  b. Centrifuge samples at 800 × *g* for 5 min and discard supernatant. Resuspend cells in 200 μL FACS buffer and transfer to a round-bottom 96-well plate. Maintain the plate on ice.
  c. Prepare antibody cocktail in 50 μL FACS buffer for each sample.
    ***Note:*** Long-lived memory T (T_MEM_) cells in peripheral blood consist of CD62L^+^CD69^−^ T_CM_ and CD62L^−^CD69^−^ T_EM_ subsets; the surface staining cocktail includes antibodies against CD8, CD45.1, CD44, CD69, CD62L and Live/Dead dye.
  d. Resuspend cell pellets in 50 μL FACS buffer containing Fc-Block (1:200 final dilution) and incubate on ice for 20 min.
  e. Add 50 μL prepared antibody cocktail directly to each well and incubate samples for 30 min on ice (2°C–8°C).
  f. Wash cells twice with 200 μL FACS buffer per well and transfer to flow cytometry tubes for acquisition.
9. Acquire samples on a BD Fortessa flow cytometer.
  a. Prepare a gating strategy in the flow cytometry global worksheet as depicted in Figure 2B.
  b. Acquire a minimum total of ≥ 1 × 10^6^ events per sample. Under our experimental conditions, we typically obtain 1–5 × 10^4^ P14 CD8^+^ T cell events per sample.

**Figure 2:**
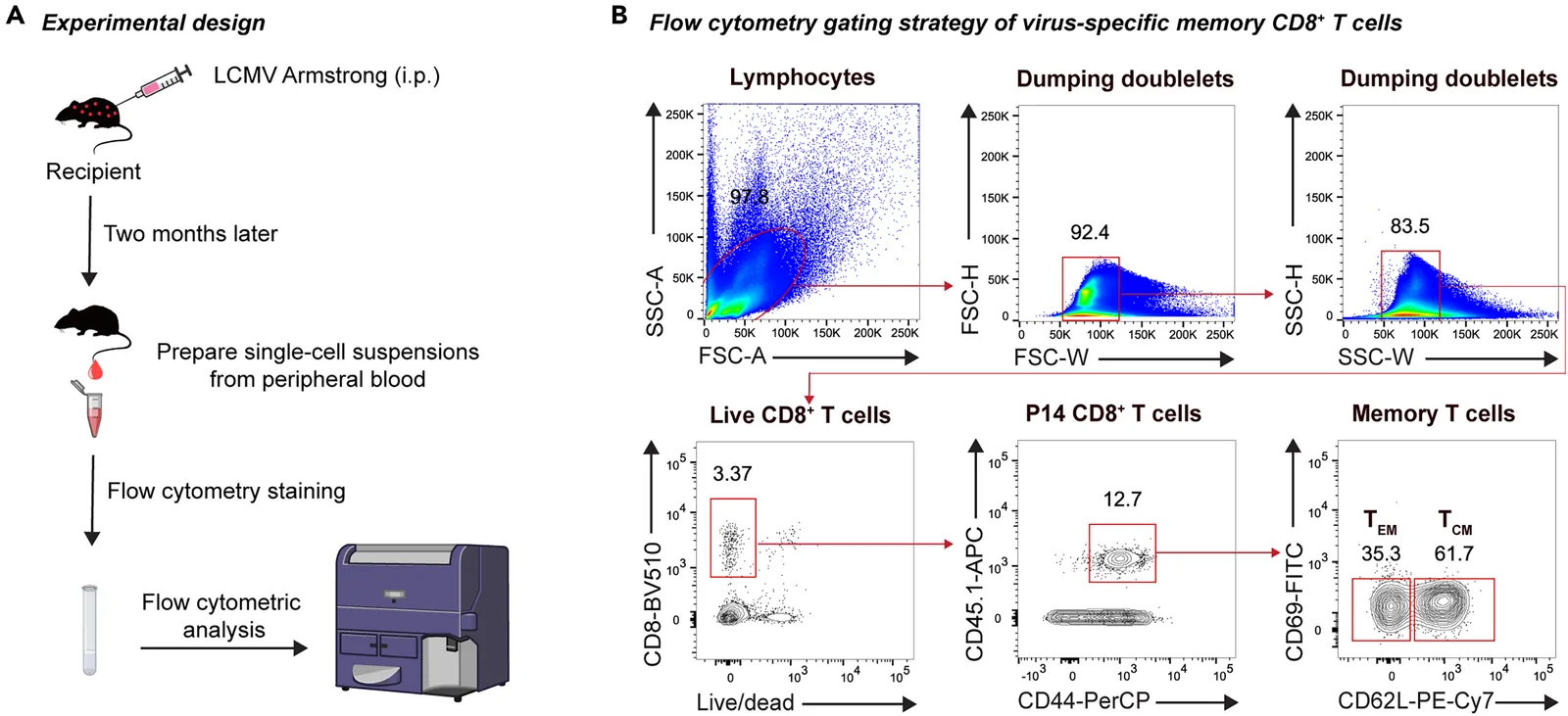
Workflow and gating strategy for flow cytometric phenotyping of LCMV-induced memory P14 CD8^+^ T cells (A) Schematic of the experimental workflow. Inject mice intraperitoneally with LCMV Armstrong, then harvest peripheral blood two months post-infection to generate lymphocyte suspensions. Stain cells with surface marker antibodies and acquire samples on a flow cytometer for subsequent immune profiling. (B) Representative flow cytometry plots showing the gating strategy of transferred P14 CD8^+^ T cells from the peripheral blood of recipients on day 60 post-infection.

### *In vitro* culture of MC38 colon adenocarcinoma cells

**Timing: 8–10 days**

This section describes routine culture for MC38 colon adenocarcinoma cells.

***Note:*** D10 medium is used for MC38 culture; full composition is provided in the recipe table within the materials and equipment section.

10. Thaw cryopreserved MC38 cells.
  a. Place a cryovial of MC38 cells in a 37°C water bath and gently agitate until cells are fully thawed.
  b. Transfer thawed cells to a 15 mL conical tube, add 5 mL pre-warmed D10 medium, and centrifuge at 300 × *g* for 5 min at 20°C–25°C.
  c. Discard supernatant and resuspend cells in D10 medium.
11. Culture MC38 cells in D10 medium for 2–3 passages prior to tumor inoculation.
  a. When cells reach 90% confluence in a 10-cm cell-culture dish, aspirate spent culture medium and gently wash cells twice with 2 mL of PBS per wash.
  b. Add 1 mL 0.25% Trypsin-EDTA and incubate at 20°C–25°C for 1 min to detach cells.
  c. Terminate trypsinization by resuspending cells in 2 mL pre-warmed D10 medium.
  d. Transfer cells to a 15 mL conical tube and centrifuge at 300 × *g* for 5 min at 20°C–25°C.
  e. Discard supernatant, resuspend cell pellets in D10 medium, and passage cells to a new culture vessel at a 1:3 split ratio.

**CRITICAL:** After passaging at a 1:3 split ratio, cells typically reach 80%–90% confluency within 48 h under standard culture conditions. 80%–90% confluency is optimal for tumor inoculation; avoid using over-confluent cells.

### Establishment of subcutaneous MC38 tumor model

**Timing: 1 day**

This section describes generation of subcutaneous MC38 colorectal tumors in recipient mice.

12. Harvest and prepare MC38 cells for inoculation.
  a. Collect in vitro-cultured MC38 cells by trypsinization as described in steps 11a-11d.
  b. Discard supernatant and resuspend cell pellets in PBS.
  c. Wash cells once with 5 mL of PBS, then centrifuge at 300 × *g* for 5 min at 20°C–25°C.
  d. Discard supernatant and resuspend cell pellets in 2 mL of PBS.
  e. Count viable cells and adjust cell density to 0.5 × 10^6^ cells per 100 μL PBS.
13. Subcutaneously inoculate MC38 cells into recipient mice.
  a. Remove fur from the right flank of recipient mice using electric clippers.
  b. Using a 1 mL syringe, subcutaneously inject 100 μL MC38 cell suspension into the shaved right flank of each mouse.

***Note:*** This tumor engraftment workflow is not limited to MC38 colon adenocarcinoma cells. This identical pipeline, including adoptive transfer, LCMV infection and tumor-infiltrating lymphocyte isolation, can be applied to other subcutaneous tumor models (e.g., B16 melanoma, CT26 colon adenocarcinoma) to characterize T_BYS_ cell populations across diverse tumor microenvironments.

### Flow-cytometric analysis of tumor-infiltrating virus-specific T_BYS_ cells

**Timing: 2 days**

This section describes isolation of tumor-infiltrating lymphocytes (TILs) and flow cytometric characterization of tumor-infiltrating virus-specific P14 T_BYS_ cells (Figure 3).

14. Euthanize tumor-bearing mice 15 days post-tumor inoculation in accordance with institutional guidelines.
15. Generate single-cell TIL suspensions from tumor tissue.
  a. Mince excised tumor tissue into small fragments with sterile scissors.
  b. Digest minced tumor fragments in 5 mL digestion solution per sample (see materials and equipment) at 37°C for 45 min with continuous agitation on a rotator.
  c. Mechanically dissociate digested tumor fragments by grinding tissue against a 70 μm cell strainer placed on top of a culture dish using the plunger end of a syringe. Add small volumes of PBS in multiple aliquots during grinding to wash dissociated cells through the strainer and generate single-cell suspensions.
  d. Isolate TILs by density-gradient centrifugation using 44% and 67% Percoll solutions (see materials and equipment for solution preparation).
  e. Resuspend the single-cell pellet in 8 mL of 44% Percoll solution. Carefully overlay this cell-containing 44 % Percoll mixture onto the top of 4 mL of 67 % Percoll solution pre-loaded in a 15 mL centrifuge tube to form a sharp density interface.
  f. Centrifuge samples at 1260 × *g* for 30 min at 20°C–25°C with the centrifuge brake disengaged.
  g. Following centrifugation, aspirate the upper debris layer and carefully collect the lymphocyte-containing interphase layer. The interphase appears as a thin, whitish-opaque band located between the 44 % and 67 % Percoll phases.

**CRITICAL:** The target lymphocyte-rich interphase is a thin whitish-opaque band; excessive aspiration will co-collect unwanted debris or Percoll.

16. Flow cytometric staining of tumor-infiltrating virus-specific T_BYS_ cells.
  a. Wash harvested cell suspension once with PBS to remove residual Percoll.
  b. Transfer TIL samples to a round-bottom 96-well plate and maintain on ice.
  c. Prepare surface antibody cocktail in 50 μL FACS buffer for each sample (see materials and equipment for the composition of the surface antibody cocktail).
    ***Note:*** T_BYS_ cells are derived from virus-induced memory P14 T cells and express canonical T_MEM_ cell subset markers; the staining panel includes antibodies against CD8, CD45.1, CD44, KLRG1, CD127, CD69, CD62L, CX3CR1, TCF-1, CD103 and Live/Dead dye. While the present protocol employs LCMV infection to generate virus-specific T_BYS_ populations, this experimental pipeline is broadly adaptable to alternative viral challenge or vaccination models (e.g., influenza virus infection, SARS-CoV-2 vaccination).
  d. Resuspend cell pellets in 50 μL FACS buffer containing Fc-Block (1:200 final dilution) and incubate on ice for 20 min.
  e. Add 50 μL prepared surface antibody cocktail directly to each well and incubate for 30 min on ice, protected from light.
  f. Wash cells twice with FACS buffer, add 200 μL fixation/permeabilization buffer (from Foxp3/Transcription Factor Staining Buffer Kit, see key resources table) per well, mix thoroughly and incubate at 20°C–25°C for 30 min protected from light.
  g. Prepare an intracellular antibody cocktail (see materials and equipment) for transcription factor staining (TCF-1 included), dilute the mixture to a final volume of 50 μL with 1× permeabilization buffer for each well.
  h. Wash cells twice with permeabilization buffer (centrifuge at 800 × *g* for 5 min for each wash step to collect loose fixed-cell pellets) and incubate with the intracellular antibody cocktail for 30 min on ice, protected from light.
  i. Wash cells twice with permeabilization buffer. Resuspend cells in FACS buffer and transfer to flow cytometry tubes for acquisition.
17. Acquire and analyze tumor-infiltrating virus-specific P14 T_BYS_ cells.
  a. Prepare a gating strategy in the flow cytometry global worksheet as depicted in Figure 3A.
  b. Acquire a minimum total of ≥ 2 × 10^6^ events per sample.

**Figure 3:**
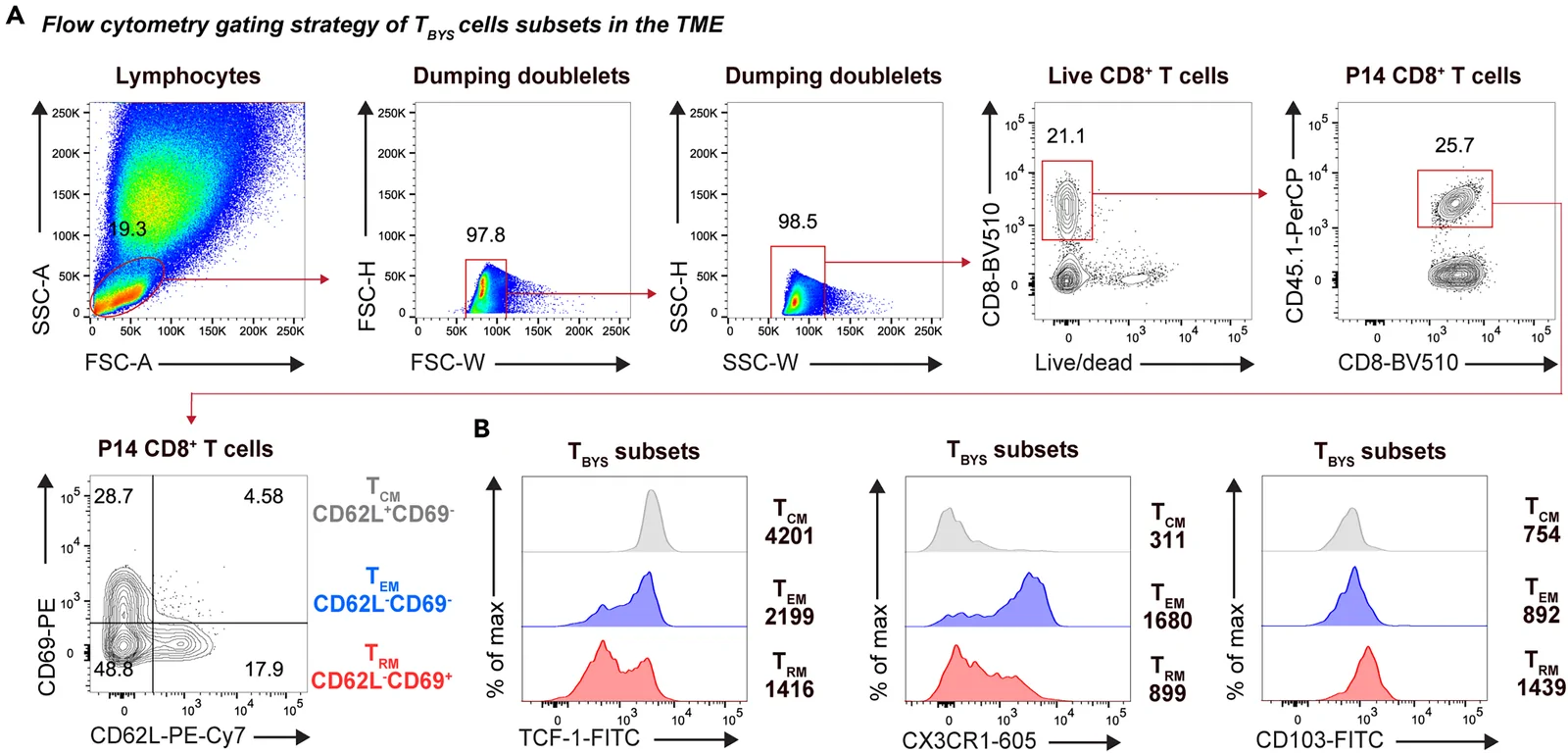
Flow cytometric analysis to characterize subsets and phenotypic profiles of tumor-infiltrating virus-specific T_BYS_ cells (A) Representative flow cytometry plots showing the gating strategy of transferred P14 CD8^+^ T cells from the TME of recipients on day 15 after tumor engraftment. (B) Histograms depicting TCF-1 (left), CX3CR1 (middle), and CD103 (right) expression in T_CM_ (gray), T_EM_ (blue), and T_RM_ (red) subsets within the TME. Numbers indicate TCF-1, CX3CR1, and CD103 mean fluorescence intensity (MFI) in each subset.

## Expected outcomes

This protocol allows long-term *in vivo* tracking of tumor-infiltrating virus-specific T_BYS_ cells. Two months post-infection, P14 T_MEM_ cells in blood primarily consisted of CD62L^+^CD69^−^ T_CM_ and CD62L^−^CD69^−^ T_EM_ cells (Figure 2B). Following tumor engraftment, TME-infiltrating P14 T_BYS_ cells comprised CD62L^+^CD69^−^ T_CM_ cells, CD62L^−^CD69^−^ T_EM_ cells and CD62L^−^CD69^+^ tissue-resident memory T (T_RM_) cells (Figure 3A). T_CM_−associated marker TCF-1, T_EM_−associated marker CX3CR1 and T_RM_−associated marker CD103 were predominantly detected in T_CM_−, T_EM_− and T_RM_−featured T_BYS_ cells, respectively (Figure 3B).

## Limitations

This protocol provides a reliable system to study tumor-infiltrating virus-specific T_BYS_ cells, with two key limitations. First, generation of virus-specific T_MEM_ cells requires more than two months, which extends the total experimental timeline. Second, this protocol does not assess responses of distinct T_BYS_ subsets to immunotherapy, which remains an important direction for future studies.

## Troubleshooting

### Problem 1

Low P14 cell purity after negative selection (related to step 3).

### Potential solution

Verify biotinylated antibody cocktail completeness (include all non-CD8 lineages). Use freshly prepared streptavidin beads and extend incubation to 45 min.

### Problem 2

Low frequency or absence of P14 cells in peripheral blood 2 months post-infection (related to step 4).

### Potential solution

Confirm P14 cell purity before transfer; ensure transfer of 5 × 10^4^ naïve P14 CD8^+^ T cells; verify LCMV titer and administer 1 × 10^5^ PFU per mouse; wash purified naïve P14 CD8^+^ T cells twice in serum-free RPMI prior to injection (see Step 4 for detailed procedure).

### Problem 3

Failed tumor engraftment with no palpable tumor outgrowth in recipient mice (related to steps 12-13).

### Potential solution

Routinely monitor MC38 growth rate in vitro prior to inoculation; if cells exhibit delayed growth, increase inoculation dose from 0.5 × 10^6^ cells to 1 × 10^6^ cells per recipient mouse.

### Problem 4

Poor viability or low yield of TILs after tumor digestion (related to step 15).

### Potential solution

Strictly limit Collagenase Type I digestion to 45 min at 37°C; use gentle mechanical dissociation.

## Resource availability

### Lead contact

Further information and requests for resources and reagents should be directed to and will be fulfilled by the lead contact, Yao Lin (lin_yao01@126.com).

### Technical contact

Requests for additional information about the protocol should be directed to Chunyang Xie (xcy1932980267@163.com).

### Materials availability

This study did not generate new, unique reagents.

### Data and code availability

There are no additional data associated with this protocol.

## Acknowledgments

This study is supported by the 10.13039/501100001809National Natural Science Foundation of China (no. 32400735 to Y.L. and no. 32300785 to X.C.), New Chongqing Youth Innovation Talent Program (no. CSTB2024NSCQ-QCXMX0030 to X.C.).

## Author contributions

Conceptualization, X.C. and Y.L.; methodology, X.C. and Y.L.; investigation, C.X., W.X., and H.W.; visualization, C.X., W.X., and H.W.; supervision, X.C. and L.Y.; writing – original draft, Y.L.; writing – review and editing: X.C. and Y.L.

## Declaration of interests

The authors declare no competing interests.
