# Supplementary material for: Protocol for analyzing tumor-infiltrating bystander CD8+ T cells in mice using adoptive transfer of P14 TCR-transgenic CD8+ T cells
Source: STAR Protoc. 2026 Oct 6;7(4):104886. doi: 10.1016/j.xpro.2026.104886 (PMC13672856; doi:10.1016/j.xpro.2026.104886)

Figure S1. Representative flow cytometry plots illustrating the gating strategy to identify naïve P14 CD8$^+$ T cells isolated from donor mice, related to Preparation steps 1-2.

Figure S1

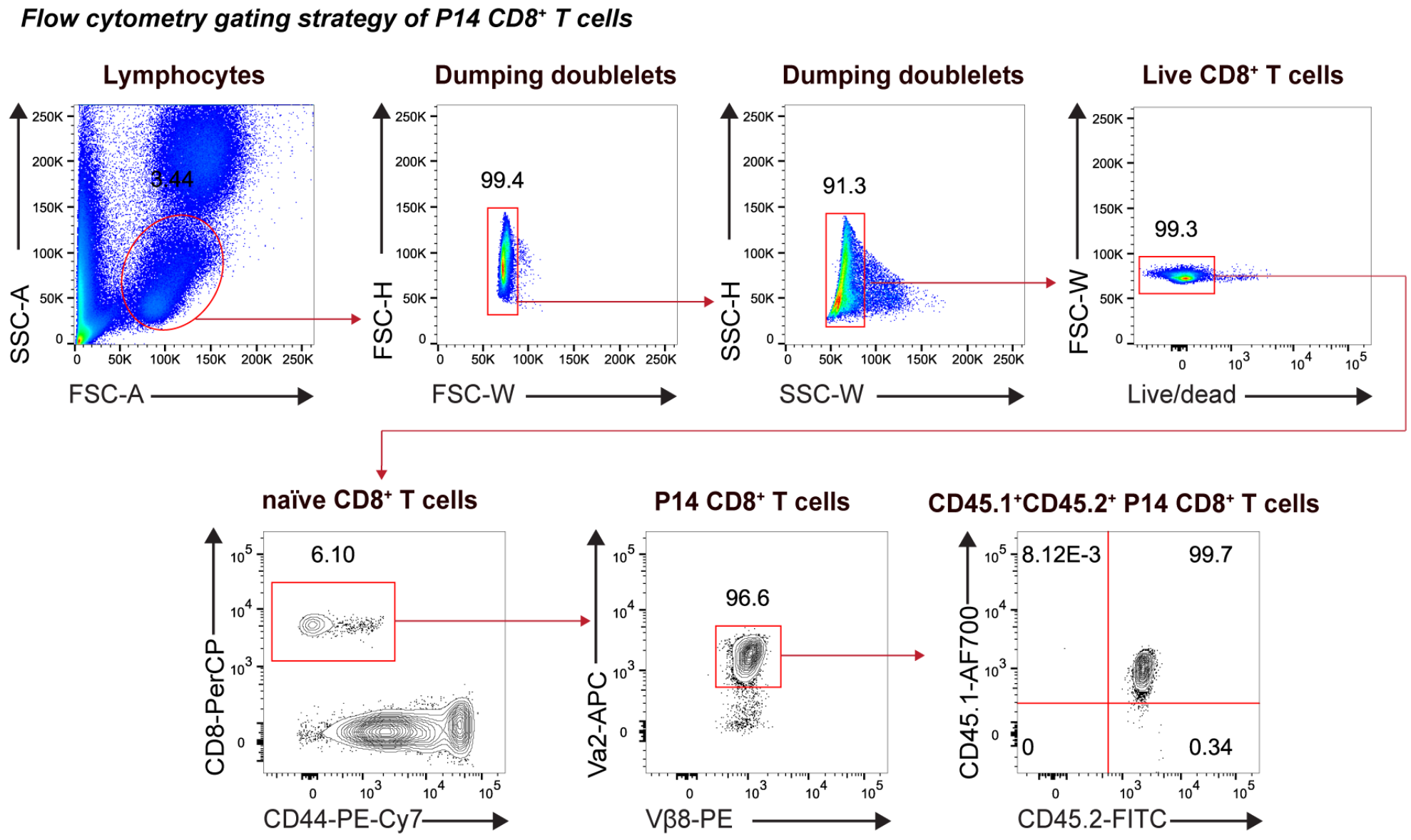

Supplement: Supplementary file 1 [file mmc1.pdf]
